# Optimizing liver disease screening and surveillance in remote Indigenous Australian communities: the SSOLID study protocol for multi-methodology research

**DOI:** 10.1136/bmjopen-2025-115620

**Published:** 2026-08-12

**Authors:** Tamara Mackean, Shane D’Angelo, Michael Larkin, Cath Brown, Greg Pratt, Patricia C Valery, Richard Woodman, Jonathan Karnon, Linda Medlin, Joshua Riessen, Melissa Carroll, Jessica Howell, Gary Jeffrey, Michael Nugent, Terrie Ivanhoe, Rochelle Menzies, Leon Adams, Greg Bird, Kirsty Campbell, Melanie Durden, Kelli Owen, Rae-Lin Huang, Iain Everrett, Cara Sheppard, Damian Riessen, Sumudu K Narayana, Alan Wigg

**Affiliations:** 1 College of Medicine and Public Health and Flinders Health and Medical Research Institute, Flinders University, Adelaide, South Australia, Australia; 2 Faculty of Health and Medical Sciences, The University of Adelaide, Adelaide, South Australia, Australia; 3 QIMR Berghofer, Herston, Queensland, Australia; 4 Central Queensland University, Rockhampton, Queensland, Australia; 5 Central Queensland Hospital and Health Service, Rockhampton, Queensland, Australia; 6 Aboriginal Health Service of South Australia, Adelaide, South Australia, Australia; 7 Royal Darwin Hospital, Darwin, Northern Territory, Australia; 8 Charles Darwin University, Darwin, Northern Territory, Australia; 9 Department of Medicine, University of Melbourne, VCCC, Parkville, Victoria, Australia; 10 St Vincent’s Hospital Melbourne, Fitzroy, Victoria, Australia; 11 Burnet Institute of Disease Elimination, Melbourne, Victoria, Australia; 12 Epidemiology and Preventive Medicine, Monash University, Melbourne, Victoria, Australia; 13 University of Western Australia, Geraldton, Western Australia, Australia; 14 Sir Charles Gairdner Hospital, Nedlands, Western Australia, Australia; 15 Umoona Tjutagku Health Service, Coober Pedy, South Australia, Australia; 16 Nganampa Health Council, Ciccone, Northern Territory, Australia; 17 Royal Flying Doctor Service of Australia (Western Operations), Perth, Western Australia, Australia; 18 Curtin University, Perth, WA, Australia; 19 University of Western Australia, Perth, Western Australia, Australia; 20 Indigenous Australian Consumer Advocate, Adelaide, South Australia, Australia; 21 Royal Darwin Hospital, Casuarina, Northern Territory, Australia; 22 Pormpuraaw Primary Health Care Centre, Pormpuraaw, Queensland, Australia; 23 Victoria University, Melbourne, Victoria, Australia; 24 Central and Northern Adelaide Renal and Transplantation Service, Royal Adelaide Hospital, Adelaide, South Australia, Australia; 25 South Australian Health and Medical Research Institute, Adelaide, South Australia, Australia; 26 Puntukurnu Aboriginal Medical Service, Newman, Western Australia, Australia; 27 South Australian Hepatology and Transplant Medicine Unit, Flinders Medical Centre, Bedford Park, South Australia, Australia

**Keywords:** Hepatology, Australian Aboriginal and Torres Strait Islander Peoples, Chronic Disease, Health Equity

## Abstract

**Introduction:**

Recognising and acting on the connection to Country as a determinant of Indigenous peoples’ well-being is necessary to improve health inequities. Indigenous Australians experience a greater burden of chronic liver disease and poorer outcomes due to ongoing impacts of colonisation across determinants of health. This is exacerbated by a gradient in health outcomes based on remoteness, lack of specialist healthcare services and barriers to access. Our research aims to explore better ways to provide chronic liver disease screening and surveillance for very remote Indigenous Australian communities using non-invasive technologies On-Country.

**Methods and analysis:**

Using an innovative combination of Indigenous and Quantitative research methodologies, this project involves 11 communities across four very remote sites in South Australia and Western Australia. The study comprises three parts: (1) site engagement with Aboriginal health services and remote communities; (2) a 12-month liver check (screening) phase and (3) a 24-month liver monitoring (surveillance) phase. The liver monitoring phase will use a stepped-wedge randomised controlled trial design where sites will have usual hepatocellular carcinoma (HCC) monitoring for a period of between 6 and 18 months and then On-Country monitoring for a period of between 6 and 18 months depending on treatment-sequence allocation. Recommended HCC monitoring involves 6 monthly liver ultrasounds and serum alpha-fetoprotein as per the site’s usual care processes, where participants travel to regional centres for liver ultrasound. On-Country monitoring will involve liver ultrasound and serum tumour markers provided On-Country every 6 months. The primary outcome is the difference in adherence to surveillance On-Country compared with usual care. In addition to statistical and health economic methods, yarning circles have been incorporated to explore participant experiences, their knowledge of liver disease and views about the On-Country monitoring.

**Ethics and dissemination:**

This study was granted ethics approval from the relevant national and state Aboriginal Health Research Ethics Committees. Findings will be reported to all participants and will be disseminated to the broader community and local health services. Translation of outcomes will be supported by key Indigenous Australian and healthcare stakeholders, including peak health bodies and consumer groups. Dissemination with the academic community will be through peer-reviewed publications and presentations at relevant conferences.

**Trial registration number:**

ACTRN12625000256471.

**STRENGTHS AND LIMITATIONS OF THIS STUDY**

- The project benefits from Indigenous Australian leadership to facilitate two-way knowledge exchange.
- This study uses a multi-methodology approach, combining Indigenous research methodology and quantitative research methodology, to design a project at the interface of knowledge systems.
- Community is at the heart of the project: local community leadership groups and Indigenous project officers at each site will ensure that local community needs and cultural practices guide the conduct of the research.
- Limitations include the challenges of working with and balancing the different priorities and needs of participating health services and very remote communities.
- The large geographic region of this project may pose logistical challenges with the implementation of the proposed research.

## Introduction

 Indigenous Australians are the First Peoples of Australia and represent long-standing knowledge systems, including about health and healing. For Indigenous peoples, culture and Country are fundamental to health and well-being, and notions of health include *‘the right to practice cultural values, beliefs and norms, including such practices that reaffirm connection to Countr*y’.^1^ In contrast to Western perspectives, Country is considered sentient and familial, with profound bounds between Indigenous peoples and all aspects of their lands, waterways, flora and fauna.^2^ Country is the basis of spirituality and the foundation of knowledge systems, social structures and cultural practices that are entwined with all living things.^3^ These relationships are vital for identity, health and well-being and the reciprocal health benefits gained from connection to Country are encapsulated in sayings such as ‘*healthy country, healthy people*’ and ‘*if you look after the country, the country will look after you*’.^4^ Holistic views of health held by Indigenous peoples around the globe are well documented.^5 6^ They include the importance of connection to Country and the interconnection of cultural and environmental aspects on both the health of the community and the well-being of the individual.^7 8^ Despite generations of cultural knowledge highlighting the importance of connection to Country for Indigenous peoples, to date there is very limited adaptation of this knowledge in implementing effective population-based healthcare for Indigenous peoples.

Dislocation of Indigenous Australians from Country and kin is associated with poorer health outcomes, and the consequences of leaving Country for healthcare include grief, a sense of loss, a loss of safety and disempowerment.^9 10^ Recent models of healthcare have focused on (re)connecting patients with their community and Country to improve health and wellbeing. These models have focused on bringing healthcare to communities via mobile facilities.^11^ Mobile dialysis units have been proven to provide much needed access to haemodialysis for rural and remote patients around the world.^12^ In Australia, there are dialysis buses providing care to Indigenous Australians On-Country, such as the Purple Truck^13^ and the Country Health South Australia (SA) dialysis bus.^14^ Similar considerations are being made in other areas of health care such as maternal care,^15^ mental health care^16^ and palliative care On-Country.^17^ These examples have demonstrated that mobile models of health care can be successfully established On-Country to meet healthcare needs for Indigenous Australians without the need to compromise spiritual wellbeing for physical health.

Liver disease, including hepatocellular cancer (HCC), is the third main contributor to the chronic disease mortality gap between Indigenous Australians and non-Indigenous Australians aged 35–74 years.^18^ Risk factors for chronic liver disease (CLD), including hepatitis B virus,^19^ hepatitis C virus,^20^ alcohol misuse,^21^ diabetes^22^ and obesity,^23^ are more prevalent in Indigenous Australians compared with non-Indigenous Australians. These risk factors are driven by ongoing colonisation and oppression, such as intergenerational and complex trauma, addictions, embedded structural disadvantage, over-incarceration, dispossession, food insecurity and denial of Indigenous sovereignty. The inequities in liver disease are even greater for Indigenous Australians residing in rural and remote communities throughout regional Australia.^24^ A large multi-jurisdictional study found the majority of Indigenous Australians with HCC (53%) lived remotely, in contrast to 3% of non-Indigenous Australians with HCC.^25^ Other key findings from this study were the identification of the most common conditions associated with HCC: alcohol misuse (54%), diabetes (48%), hepatitis B viral infection (25%) and the frequent co-existence (51%) of these conditions.

Compounding the high prevalence of CLD in Indigenous Australians living in remote communities is limited access to specialist care and diagnostics. Our own clinical experience and recent clinical audit data demonstrated very low (7%) use of HCC surveillance (both ultrasound and alpha-fetoprotein (AFP)) in SA Aboriginal community-controlled health service (ACCHS) settings.^26^ A major factor contributing to low use was the substantial distance and costs associated with travel to regional centres to access radiology services. There is also evidence demonstrating the impact of lack of access to life saving, evidence-based therapies for Indigenous Australians with liver disease, including liver transplantation and curative treatments for HCC.^25 27^ A lack of knowledge among primary care clinicians also contributes to inequities, with limited inclusion of CLD in current primary care guidelines, such as those by the Central Australian Rural Practitioners Association.^28^

Remote Indigenous Australian communities could benefit significantly from the recent technological advances in testing for liver disease to overcome geographical barriers to best practice medical care. Serum-based fibrosis tests (Fibrosis-4 (FIB-4), Hepascore), complemented by mobile FibroScan® devices (liver elastography) can be performed in remote communities and can accurately screen for compensated advanced chronic liver disease (cACLD) or cirrhosis in populations at risk of disease without the need for an invasive liver biopsy.^29–31^ Miniaturisation of ultrasound has enabled high quality liver ultrasound to be performed in community settings, using portable equipment^24^; this can improve the uptake of HCC surveillance guidelines, a current standard of care that has shown to reduce mortality through detection of early, curable HCC.^32 33^ The incorporation of liver elastography into ultrasound machines now allows for simultaneous ultrasonography and elastography in one mobile device. Furthermore, recent advances in serological tumour marker testing (GAAD score) for HCC screening and early detection have the potential to enable accurate, non-invasive and repeatable HCC screening in remote communities,^34 35^ potentially removing the need for ultrasound surveillance in lower-risk patients.

Efforts to improve outcomes of CLD and HCC in Indigenous Australians must therefore address barriers to healthcare access for remote communities. These efforts need to generate insights and evidence into enablers and barriers to CLD screening and surveillance uptake through best practice in Indigenous health research. The objective of this study is to evaluate new ways of CLD screening and surveillance in very remote Indigenous Australian communities using non-invasive technologies On-Country. Specifically, this study aims to assess the feasibility, acceptability and effectiveness of On-Country liver disease screening and surveillance in very remote Indigenous Australian communities.

### Governance

Indigenous leadership is critical for this research to ensure that it properly centres Indigenous knowledge systems and ways of working. This will allow us to incorporate the context, preferences and needs of each site into the conduct of the research. The project has a robust Indigenous governance structure with a First Nations Investigator Group (FNIG) and a Community Leadership Group (CLG). The FNIG consists of all Indigenous Australian Chief Investigators (CIs) and Associate Investigators (AIs) (10 investigators) and will oversee Indigenous governance of the project as a whole, guide the project to ensure rigour in the application of Indigenous Research Methodologies (IRM) and support the functions of the CLG. The project has Indigenous Australian investigators in leadership roles in all aspects of the project, as well as having the CLG for local leadership. A CLG will be established at each site and may include regional primary healthcare staff, community elders and local community members, who will be the primary mechanism for community liaison and local leadership with support from the regional study team and the project team. The regional study team will include a principal investigator (PI) and Indigenous Project Officer (IPO) who will be involved in the conduct of the project at each site, have direct communication with the CLG and participants and will be supported by the project team. The project also has strong clinical governance with representation from a range of sites, professional groups and key Indigenous groups on the steering committee, along with a Data Safety Monitoring Board and a medical monitor (Hepatologist) for clinical guidance.

## Methods and analysis

### Positionality statement

The author group is a mixture of Indigenous and non-Indigenous researchers across all career stages. TM is a Waljen woman and public health medicine physician; SDA is a Kokotha man and Indigenous health researcher; ML is a Kokatha man and Indigenous health researcher; CB is a Quandamooka woman and Indigenous health researcher; GP is a Quandamooka man and Indigenous health researcher; PCV is a non-Aboriginal woman and medical epidemiologist; RW is a non-Aboriginal man and biostatistician; JK is a non-Aboriginal man and health economist; LM is a Kalkadoon woman and Indigenous health researcher; JR is a Yankunytjatjara man and blood-borne virus programme officer; MC is a Wiradjuri woman and the first Aboriginal gastroenterologist and hepatologist; JH is a non-Aboriginal woman and hepatologist and clinical researcher; GJ is a non-Aboriginal man and hepatologist and clinical researcher; MN is a non-Aboriginal man and primary care physician; TI is a non-Aboriginal woman and a chronic disease coordinator; RM is a Māori (Te Aitanga-a-Māhaki and Ngāti Kahungunu) woman from Aotearoa New Zealand and senior Indigenous health researcher; LA is a non-Aboriginal man and hepatologist and clinical researcher; GB is a Kokotha man and consumer advocate with lived experience of liver disease; KC is a non-Aboriginal woman and hepatologist; MD is a non-Aboriginal woman and health worker; KO is a Kaurna, Narungga and Ngarrindjeri woman and Indigenous health researcher and cultural advisor at CNARTS; RLH is a non-Aboriginal woman and primary care physician; IE is a non-Aboriginal man and a chronic disease coordinator; CS is a non-Aboriginal woman and primary care physician; DR is a Yankunytjatjara man and health worker; SN is a non-Aboriginal woman and research scientist and AW is a non-Aboriginal man and hepatologist and clinical researcher.

### Patient and public involvement statement

The Screening and Survillance of Liver Disease in Indigenous Australians (SSOLID) CI and AI group includes people with lived experience (patients) who have contributed to the study design and submission of this project to the funding body. AIs KO and GB provided patient perspectives in the development of the research question, as well as contributing to the study resources. Phase 1 of the project focuses on working with each participating community (public) to understand their key health priorities and identify shared interests regarding CLD care. Based on these discussions, study documents have been translated into local Indigenous languages and consent videos created to support recruitment. The study has also established a CLG at each community, as part of project governance, and these groups will inform the study team on community context and how project results are disseminated to their communities.

### Methodology

**Figure 1:**
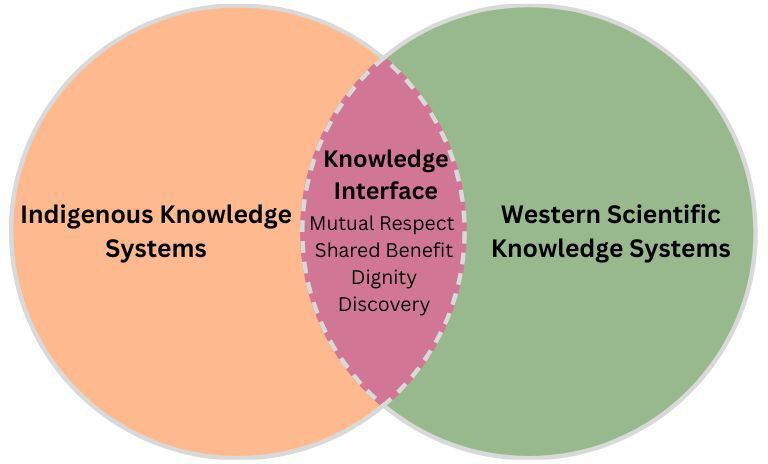
Visual representation of the interface between Indigenous and Western scientific knowledge systems (based on Durie, 2005).

Best practice in Indigenous health research includes fostering Indigenous leadership in projects, engaging with Indigenous communities throughout the research, using Indigenous research paradigms where appropriate, taking a strength-based approach in research activities, ensuring benefits to those involved and translating research findings into policy and practice.^36 37^ In this project, we have used the interface of knowledge systems as described by Durie^8^ to bring together Indigenous ways of knowing, being and doing with Western biomedical epistemology, ontology and axiology. Specifically, this means bringing together IRM (relationality and data sovereignty) with Quantitative Research Methods (QRM) (clinical trials and health economics) in a non-hierarchical manner to produce quality evidence to transform health services and systems (figure 1, based on Durie 2005).^8^

Working at the interface will involve building the capacity of the entire research team to engage with both Indigenous concepts of health and healing and Western biomedical approaches to disease and treatment. We will do this through face-to-face and online sessions to deepen our knowledge of the Country and the sites at which the research is being undertaken. We will also focus on building knowledge about IRM and foster respectful and open discussions between the researchers. As such, the SSOLID study will merge different ways of working and create strong partnerships among researchers, with research sites and with key stakeholders to facilitate sustainable change in current healthcare practice and policy. This will contribute to national efforts to reduce the gaps in liver disease care and outcomes for Indigenous Australians.

### Study design and settings

**Figure 2:**
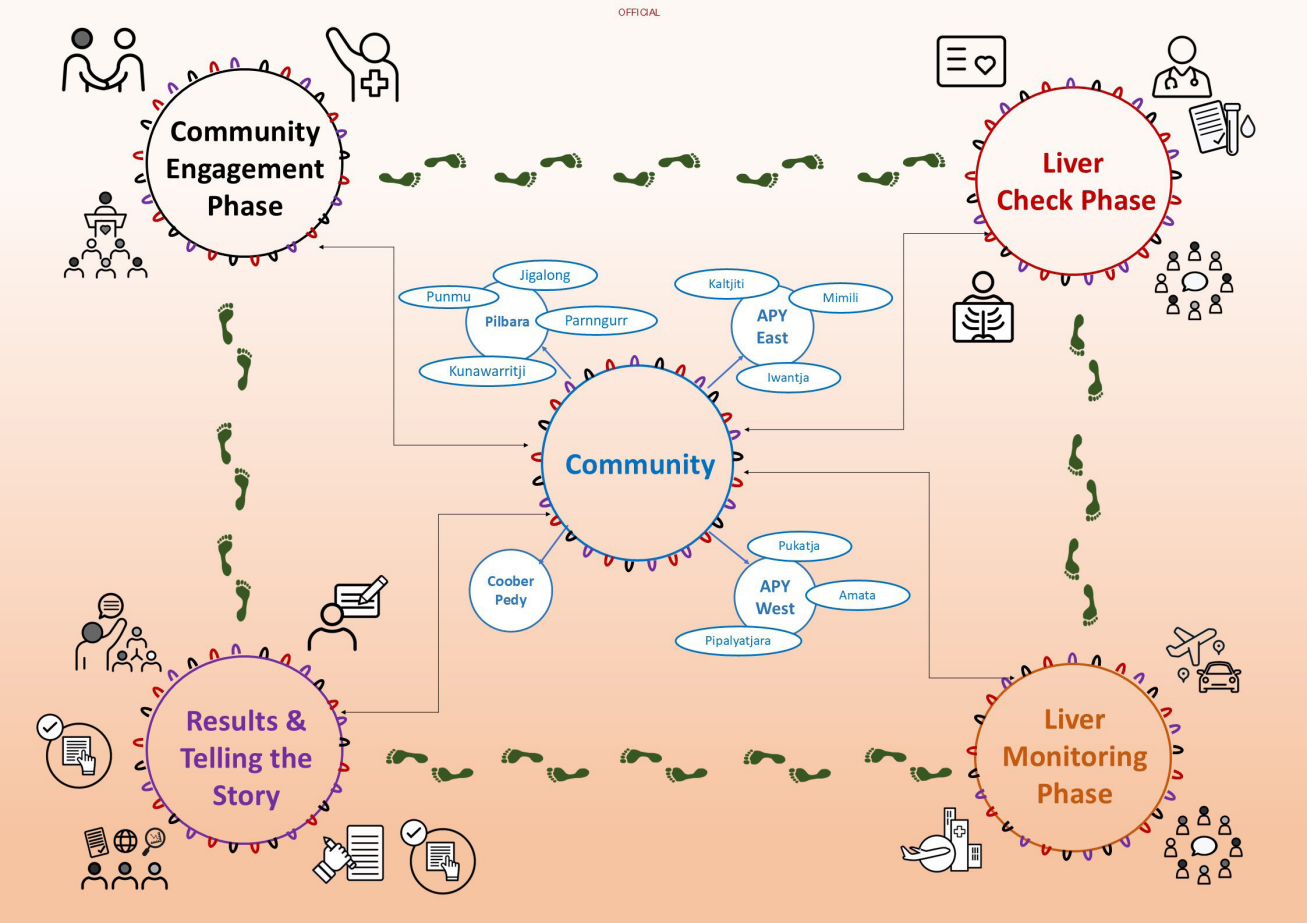
Screening and Suveillance of Liver Disease in Indigenous Australians (SSOLID) study design.

Durie outlines four main principles that underpin working at the knowledge interface^8^: mutual respect, shared benefits, human dignity and discovery, and these principles will guide the application of our methods. This study will employ a multi-method approach by using IRM of relationship building, yarning and deep listening alongside QRM of randomised clinical trial (RCT) statistical analyses and health economic analyses. The study is divided into three phases: (1) the site engagement phase, (2) the liver check (screening) phase and (3) the liver monitoring (surveillance) phase (figure 2). The liver monitoring phase will use a stepped-wedge RCT design to assess the feasibility and adherence to On-Country study design surveillance. The SSOLID project will run from October 2023 to September 2029, with Phase 1 occurring from January 2024 to June 2024, Phase 2 occurring from July 2025 to September 2026 and Phase 3 occurring from October 2026 to October 2028.

This study will take place in four very remote (Modified Monash 7) Indigenous Australian study sites (11 communities) across SA and Western Australia (WA). The sites are the Anangu Pitjantjatjara Yankunytjatjara (APY) Lands East in SA, including Mimili, Kaltjiti and Iwantja communities; the APY Lands West in SA, including Pukatja, Amata and Pipalyatjara communities; Coober Pedy in SA and the East Pilbara region in WA, including Jigalong, Parnngurr, Punmu and Kunawarritji communities. The research team will work with the local ACCHSs at each site to implement the study and ensure it meets local community needs, and this partnership will be formalised via a clinical trial agreement.

### Yarning

The concept of yarning in research has arisen from a need to connect with Indigenous concepts of health and healing through culturally appropriate means.^38^ Yarning is not just talking; rather, it involves talking, listening, asking questions, reflecting on answers, responding to what is being said and allowing moments of silence for deep thinking. It is a fundamental aspect of Indigenous knowledge creation and maintenance, including the way in which information is shared in multisensorial ways. Knowledge is contained in stories that are embodied in Country, flora, fauna and people; consequently, yarning is place-based and highly relational.

Different types of yarning are used in research,^39^ including social yarning, which allows participants and researchers to build rapport, research yarning which allows discussion on the research topic, collaborative yarning which can occur between researchers during analysis as well as between researchers and communities during engagement and therapeutic yarning which acknowledges the need for empathic approaches when working with those who have suffered colonial trauma. We intend to use yarning (at the end of Phase 2 and Phase 3) to engage with research sites to develop a rich understanding of the specific remote context for each site in order to meaningfully position quantitative findings and thus promote effective translation. We will also use yarning to collect data on experiences of both screening and surveillance, and yarning guides will be used to facilitate these. The yarning guides will include a relational yarn component (rather than a social yarn) because kinship systems are an integral part of Indigenous culture, and in order to engage and speak with someone, both researchers and participants need to understand how they are ‘related’ to one another. Only then can rapport be built and information exchanged.

### Phase 1: Community Engagement

Research activities at each site will incorporate relationality to ensure the research responds to community needs. A relationality worldview understands that no one and nothing exists in isolation, but rather all people, sociocultural systems and the environment are interconnected.^40^ In this study, forming relationships with the local ACCHSs and conducting engagement visits with Elders and other relevant stakeholders at our study sites is a foundational component of the project. This includes presenting to ACCHS Board members and seeking permission to undertake further relationship building and study development On-Country. Indigenous Australian CIs conducting these visits will discuss the proposed research, seek feedback on study implementation, including site enrolment in the Australian Teletrial Program, and the possible impacts of the study for the community and local health service. The Australian Teletrial Program supports the conduct of clinical trials in rural, regional and remote areas through per participant funding to health services to cover costs not included in the project budget. These visits provide opportunities for the research team to identify shared interests with the local community and ACCHSs, and build strong relationships with each of the study sites.

Training sessions will be hosted by the study investigators at each study site to build understanding of liver disease within communities, as well as upskilling and building capacity of primary healthcare staff in CLD management. Relevant staff from each participating site will be recruited internally, while local IPOs will be recruited at each participating site to assist with the implementation of the project, have direct communication with participants and help provide the researchers with local context. The training sessions will focus on liver disease and project-specific activities. The site PI and IPO will also be trained in conducting a FibroScan® assessment. Training materials and resources will be developed in conjunction with the local health services and CLGs, and translated into relevant Indigenous languages by certified translators. The local team will continue to be supported throughout the project during formal fortnightly meetings to manage day-to-day conduct of the study and discuss any challenges.

### Phase 2: Liver Check (Screening)

The liver check phase has a screening design (12 months) and will identify people with clinically significant liver disease eligible for Phase 3. In the liver check phase of the project, the site PI, IPO and support ACCHS staff will review the primary healthcare patient databases to ascertain participants who meet study inclusion criteria for screening (table 1). Recruitment will be via invitation only and led by the site PI and IPO. Participating study sites will aim to screen approximately 100 participants at their site. The liver health check will involve a review of the participant’s medical history and a blood test for liver function and serum fibrosis assessment (FIB-4 and Hepascore) and will be completed On-Country at the local ACCHS. A positive indirect (FIB-4≥1.3) or direct (Hepascore ≥0.6) fibrosis test result will trigger a FibroScan® assessment. The FibroScan® assessment will be done On-Country by the local site team. All participants will be recompensed for their time and contribution to the study.

**Table 1:** Inclusion and exclusion criteria for the liver check phase

Inclusion criteria	Exclusion criteria
Aboriginal and/or Torres Strait Islander descent Aged ≥25 years Defined by the prevalence of liver disease at lower age groups and the aim to focus the study on the population who will benefit the most. Able to provide informed consent Has at least one risk factor for liver disease: Body Mass Index (BMI) >30 Current chronic hepatitis B virus Hepatitis C virus (current or past infection) Type 2 diabetes Unknown hepatitis B vaccination status Current or past alcohol misuse (an Alcohol Use Disorders Identification Test-C score of≥3 for women and ≥4 for men or clinical notification of alcohol misuse) Abnormal liver function tests (above normal range)	Unable to provide informed consent <25 years of age No known risk factors for liver disease

A FibroScan® result of >12 kPa, or if a participant has chronic hepatitis B and is >50 years of age, will trigger eligibility into Phase 3 of the study. Participants with a FibroScan® result of >10 kPa will have their overall risk profile for HCC reviewed by the medical monitor to determine entry into Phase 3 of the study. Participants with higher clinical risk factors (age>50 years, multiple liver risk factors or thrombocytopaenia) will be entered into the HCC monitoring phase with this lower liver stiffness measurement cut-off of >10 kPa.^41^ In the event, a FibroScan® assessment is unable to be performed on a participant with elevated fibrosis tests, a Hepascore≥0.8 or a liver outcome score for HCC risk≥10% at 5 years will be used as the cut-off to identify participants with cACLD. Based on conservative estimates of a prevalence of significant liver disease in 20% of screened participants, we anticipate that each site will be able to recruit approximately 10 participants for Phase 3 of the study assuming a consent rate of 50%.

### Phase 3: Liver Monitoring (HCC surveillance)

**Figure 3:**
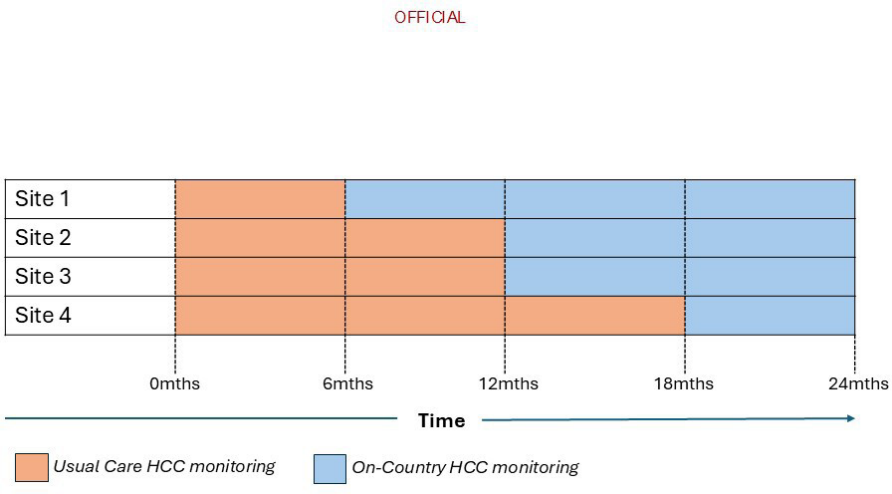
Stepped wedge randomisation of study sites. HCC, hepatocellular carcinoma.

The liver monitoring phase is a 24-month stepped-wedge RCT to compare two models of HCC surveillance (usual care vs On-Country monitoring) for Indigenous Australians who live in very remote communities. The stepped-wedge RCT design is both justifiable and optimal—relative to a conventional parallel clustered trial—in very remote Indigenous Australian community settings because of the small number of sites and because it does not exclude anyone from access to treatment, which could further exacerbate inequities. All sites will have between 6 months and 18 months of usual care before commencing the On-Country HCC surveillance period at different time intervals, which will continue for 6–18 months depending on randomisation allocation (figure 3).

#### Inclusion/exclusion criteria

Participants identified in the liver check phase as having clinically significant liver disease will be eligible to take part in this phase (table 2). Nil exclusion criteria.

**Table 2:** Inclusion and exclusion criteria for the liver monitoring phase (HCC surveillance)

Inclusion criteria	Exclusion criteria
Participants with compensated advanced chronic liver disease (cACLD) or cirrhosis identified during the screening phase by any of the following: Clinically diagnosed on the basis of clinical and radiological features, or Liver stiffness score>12 kPa, or Hepascore≥0.8 or liver outcome score for HCC of ≥10% at 5 years (if FibroScan® unavailable) Liver stiffness score>10 kPa if participants have higher clinical risk factors (age>50 years, multiple liver risk factors or thrombocytopaenia). Participants with chronic hepatitis B virus infection, without cirrhosis, who are >50 years	Nil

HCC, hepatocellular carcinoma.

#### Randomisation

The four participating sites (groups) will be the unit of randomisation in the 24-month stepped-wedge RCT design. The sequence in which these four sites cross over from the 'usual care’ control period to the 'On-Country’ intervention period will be randomly determined. An independent statistician with no involvement in participant recruitment will generate the random allocation sequence using a computer-based random number generator with statistical software (Stata, StataCorp, US). Each site will begin the trial receiving usual care surveillance for a minimum of 6 months, and the randomisation sequence will determine at which 6-month time point each site transitions from usual care to the On-Country surveillance. The generated allocation schedule will list the 24-month care sequence for each site (A, B, C and D) and will be securely stored and concealed from the core study team until the point of implementation to prevent any potential bias. The four sites will be assigned a site code (A, B, C or D) by the investigators prior to receiving the allocation schedule from the biostatistician. Given the small number of sites, stratification will not be used.

#### Usual HCC monitoring (control)

Usual HCC surveillance (control group) will involve participants receiving 6-monthly liver ultrasound and serum AFP tests as per their site’s usual care processes. This will involve participant travel to the closest regional imaging service for liver ultrasound, with each scan reviewed and reported by a qualified radiologist.

#### On-Country HCC monitoring (study intervention)

On-Country surveillance will involve participants receiving 6-monthly liver ultrasounds and serum tumour (AFP and GAAD) tests On-Country. The On-Country liver ultrasounds will be performed by a specialist ultrasonographer using a mobile ultrasound (Sonosite PX Ultrasound System) for detection and assessment of liver lesions, with each scan reviewed and reported by a qualified radiologist. The blood draw for the serum tumour tests will be conducted at the local ACCHS. The duration of the monitoring period will vary as per the stepped-wedge design and allocation schedule.

Surveillance ultrasound will be considered positive if it detects a liver lesion ≥10 mm in maximal diameter and combined with elevated serum AFP as per current guidelines. If an HCC diagnosis is suspected, participants will be referred to the existing care pathways for diagnosis, treatment and management.

##### Sample size and power calculations

The study is powered to detect a clinically meaningful difference in adherence to HCC surveillance. With four sites and a minimum of 10 participants per site (total n=40), the study will have 80% power to detect an absolute difference of 24 percentage points in adherence between the control and On-Country surveillance periods (eg, an increase from 10% in the control periods to 34% in the intervention periods). This calculation is based on a two-sided alpha of 0.05 and assumes four measurement periods over 24 months (one during the initial baseline control period for all sites, followed by three 6-monthly measures). It further assumes an intra-class correlation coefficient of 0.05 to account for the correlation between participants within the same site. To ensure a final analytic sample of at least 40 participants, we will recruit 45 individuals to allow for a potential attrition rate of approximately 10%.^42^ The overall 24% difference in adherence between care types is a conservative estimate of anticipated improvement, based on our published experience of 64% improvement in 2-year surveillance following a clinical practice improvement programme in a metropolitan hospital setting (44). The estimated 10% adherence in the control period is also conservative given much lower values described in current literature.^43^

## Data collection

### Consent

Participant information and consent forms will be in English and in relevant Indigenous languages (example in online supplemental file 1). Informed consent will be obtained via the site PIs and IPOs and will include an audio-visual presentation of participant information. Consent will be obtained for involvement in the study, for taking samples, for recording yarns and for accessing Services Australia data for economic analysis. Data collected will be used expressly for approved activities exclusively for this research project. Specified consent will be obtained from participants, and no data collected will be used for a secondary or unspecified purpose.

### Quantitative data

Participant sociodemographic and clinical information will be collected at the Liver Check phase (prior to randomisation), namely age, sex, relevant medical history (eg, liver disease risk factors), blood test results (including liver function tests) and serum fibrosis assessments (FIB-4 and Hepascore). Outcome data (adherence to HCC surveillance) will be collected prospectively during the liver monitoring phase, including dates liver ultrasound, AFP and GAAD tests are performed.

### On-Country liver ultrasound data

The adequacy of the On-Country ultrasound exam for the exclusion of liver lesions, including HCC, will be obtained using standard scoring systems of visualisation.^44^ The following visualisation criteria will be applied: (i) no or minimal limitations, defined as any limitations that are present but unlikely to meaningfully affect the sensitivity in the detection of masses; (ii) moderate limitations, defined as the presence of any limitations that may decrease the sensitivity of detecting small masses and (iii) severe limitations, defined as any limitations that significantly lower the sensitivity of focal liver observations.^45^

### Yarning data

Our research yarning will focus on understanding liver disease, experiences of different models of care and costs incurred by participants, and what participants think works well in their communities when it comes to accessing liver health checks and monitoring. Yarns will be conducted at the end of the liver check and liver monitoring phases. Additional yarns will be conducted with site-based health service staff to explore experiences and perspectives relating to (i) current liver care at the health service and any barriers to providing care and (ii) changes to the service after provision of the study education and availability of On-Country monitoring. We will use the site PIs and IPOs, and community networks, to recruit participants for these yarns. We will have recruitment information in both English and relevant Indigenous languages that can be distributed through these networks and displayed in common areas within each community. Yarns will be undertaken by Indigenous Australian investigators and will be offered in groups as well as one-on-one yarns to prioritise participants’ preferences and comfort. We aim to yarn with 6–8 participants at each community at the end of each study phase. Guidelines for all involved in the yarning circles around confidentiality and information sharing within the sessions will be set up by each CLG and will be explicit and discussed prior to the session commencing. All participants will be recompensed for their time and contribution to the yarns.

## Data analysis

**Figure 4:**
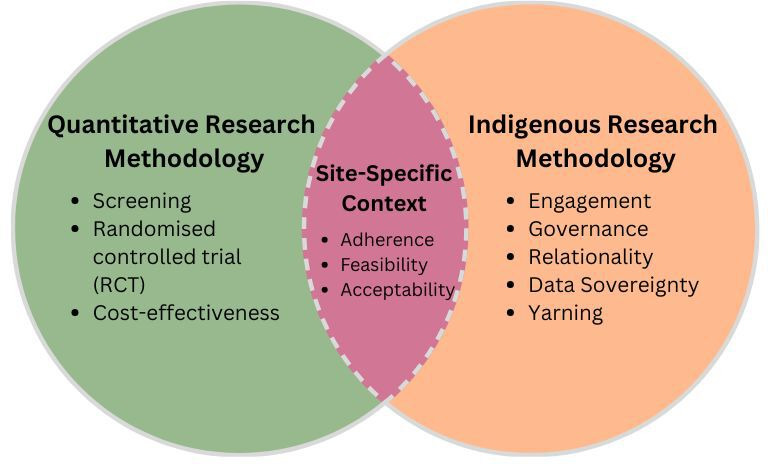
SSOLID methodology.

This project will incorporate a multi-methods approach to data analysis with integration between IRM and QRM analysis to ensure outcomes have a site-specific context and are within the knowledge interface (figure 4). The primary outcome is the difference in adherence to HCC surveillance between usual care surveillance and On-Country surveillance. Adherence to HCC surveillance is defined as the proportion of participants in which HCC surveillance was undertaken. Secondary outcomes include the acceptance and feasibility of the On-Country liver health model, the cost-effectiveness of the On-Country model compared with usual care, and the adequacy of liver ultrasound examination.

### Primary outcome

The primary analysis will be conducted on a modified intention-to-treat (ITT) basis, where all participants that are randomised are included in the analysis and are assigned to their original treatment according to the randomised crossover schedule, regardless of treatment received. Adherence to HCC surveillance will be operationally defined as a binary variable and recorded for each participant during each 6-month (±2 months) study period. A participant will be considered 'adherent’ (coded as 1) if they complete both a liver ultrasound and the required serum biomarker tests within that period; otherwise, they will be 'non-adherent’ (coded as 0). Analysis of the binary adherence outcome will be performed using a generalised linear mixed-effects model (GLMM) with a logit link function. The model will include fixed effects for the treatment (usual care vs On-Country), time (as a 6-month categorical variable) and time x treatment. To account for the clustered data structure, random intercepts for site and for participant (to account for repeated measures on individuals and for repeated measures within each site). The primary intervention effect will be reported as the estimated marginal mean difference in proportions (95% CI) for On-Country versus usual care surveillance. We will also report the odds ratio (95% CI) for adherence for On-Country versus usual care surveillance. Missing outcome data will be investigated for patterns, and, if assumed to be missing at random, we will conduct a sensitivity analysis using a GLMM with multiply imputed datasets to ensure our findings remain robust. Subgroup analyses by site, gender, age and liver aetiology will be conducted depending on the final number of participants recruited.

### Secondary outcomes

Secondary endpoints will evaluate the feasibility, acceptability and clinical utility of the On-Country model. The model’s feasibility and acceptability will be assessed through the collaborative analysis of yarning circles conducted with participants and staff. A comprehensive economic analysis will compare the cost-effectiveness of the On-Country model to usual care. Finally, the clinical adequacy of the portable liver ultrasounds will be determined using standardised visualisation scores.

#### Yarning

The analysis for the yarning circles involves Indigenous data analysis rather than qualitative analysis. The data component from yarning is story and our unit of analysis will accordingly be storylines, rather than ‘themes’.^39 46^ Yarning circles will be audio recorded and supplemented by researcher notes taken during the yarns. A ‘listening-in’ process will be used whereby the Indigenous Australian investigators will collectively listen to the audio recordings of the yarning circles. The audio recordings and the transcription of the yarning circles are considered paired data sources. The ‘listening-in’ process facilitates appreciation of the meanings of silences, pauses and Indigenous Australian language use. Listening in also returns data collectors to the time of data collection, and this assists with recall of non-verbal communication and other body language, which then provides greater insight for analysis. A transcript uncoupled from the audio recording is considered to be incomplete data which leaves analysis at risk of being one-dimensional. A collaborative approach to analysis will be undertaken, which allows for different Indigenous Australian interpretations of the data in the early stages of analysis to maintain the accuracy and integrity of Indigenous Australian participant voices. It also provides an opportunity to identify potentially sensitive elements of yarning circles that require appropriate custodianship and should not be transcribed but still can be included in analysis as non-textual data.

#### Economics analysis

Generalised linear mixed models will be used to compare differences in costs between the surveillance and control steps of the trial, using the same fixed and random effects as specified for the outcomes analysis. The primary cost-effectiveness endpoint is the incremental cost per additional eligible individual undergoing surveillance, which will be estimated by dividing the difference in the total costs of surveillance across the surveillance and control steps of the trial by the difference in the surveillance prevalence. To represent parameter uncertainty, non-parametric bootstrapping with replacement will be undertaken to generate 5000 bootstrap replicates of the cost and outcome variables. The results of a recently published modelled economic evaluation of 6-month HCC surveillance will be used to extrapolate the effects of observed effects of the intervention on surveillance. In people with cirrhosis, this study reported that 6-monthly HCC surveillance can increase early-stage diagnoses to up to 81% and reduce HCC mortality by 22%.^47^

#### Liver ultrasound adequacy

Descriptive statistics will be used to report the adequacy (using the 3-tiered scale previously described) of On-Country liver ultrasound, as ultrasound is expected to be challenging due to the anticipated frequent prevalence of obesity and non-fasting state in study participants.^48^

## Ethics and dissemination

### Ethics

This study has been approved by the following ethics committees: Australian Institute of Aboriginal and Torres Strait Islander Studies (AIATSIS) Research Ethics Committee (REC-0837), South Australian Aboriginal Health Research Ethics Committee (AHREC) (04-24-1147), Western Australian Aboriginal Health Ethics Committee (WAAHEC) (HREC1376) and the Southern Adelaide Clinical Human Research Ethics Committee (SAC HREC) (2024/HRE00238). Approvals have also been obtained from the Pilbara Aboriginal Health Research Alliance (PAHRA) and the Services Australia External Request Evaluation Committee. The guidelines from the Australian Institute of Aboriginal and Torres Strait Islander Studies for ethics research in Indigenous studies^49^ and the National Health and Medical Research Council of Australia guidelines for ethical conduct in Aboriginal and Torres Strait Islander health research will be followed.^50^

### First Nations Data Sovereignty

First Nations data sovereignty requires input, benefit and ownership from First Nations people.^51^ This project positions First Nations researchers at the forefront of the study. The cohort of First Nations CIs meets separately as a group, as well as having working groups covering areas of data sovereignty, yarning and translation and policy, to name a few. All working groups are led by First Nations CIs and supported by the project team. The concepts for sovereignty that will be used in this project include control and protection of data systems, dissemination of data at community and national levels, data that empower sovereignty and self-governance, data that put Indigenous people in control of how they are used, and data that are protecting and respecting Indigenous benefits.^52^ The study data will be co-owned by Flinders University, SALHN and each of the health services participating in the study, and all requests for access to data will be considered by the Data Sovereignty and Evaluation Group. All hardcopy, de-identified study data will be stored securely at each ACCHS before being securely transferred to the research team using Microsoft SharePoint. All electronic, de-identified data will be stored on a password-protected, secure server at the lead site (Flinders Medical Centre). Audio recordings and transcription of yarns will be stored on a password-protected server at Flinders University. Access to the de-identified study data will be restricted to study investigators involved in data analysis. The FNIG will also serve as the first point of contact for discussion of project results and the interpretation and reporting of those results, in keeping with the principles of Indigenous Australian data sovereignty.^53^ Data will also be reviewed by non-Indigenous Australian investigators with specific discipline expertise and then presented to the whole project team at study meetings as well as to sites and the CLG.

### Dissemination and Translation

A community-friendly summary of the final results will be provided to participants and the broader community along with visual representation of findings and a video summary. A de-identified summary will also be shared with the local health services. Due to the small nature of the communities participating, site-specific reports will not be provided to maintain participant confidentiality. Findings will also be shared in peer-reviewed publications and presentations at relevant peak national and international conferences. Translation of outcomes will be supported by key Indigenous Australian and healthcare stakeholders, including peak health bodies and consumer groups. The translation and policy working group will present findings to relevant policymakers in federal and state government jurisdictions to facilitate translation of findings into sustainable On-Country HCC monitoring across all remote and very remote Indigenous Australian communities.

### Expected outcomes and significance

The SSOLID study, in partnership with participating ACCHSs, will evaluate the feasibility, acceptability and effectiveness of providing liver healthcare On-Country to Indigenous Australians living in very remote communities. It aims to address and overcome current knowledge gaps and access barriers to good liver health by creating a mobile model of care that brings high-quality liver care to Country. By working at the interface of Indigenous and Western knowledge systems, we aim to produce evidence that is both clinically and culturally grounded. Ultimately, this research has the potential to inform policy, practice and On-Country models of care for better healthcare access and health outcomes for Indigenous Australians living in rural, remote and very remote Australia.

## Supplementary material

10.1136/bmjopen-2025-115620 online supplemental file 1

10.1136/bmjopen-2025-115620 online supplemental file 2
