# Supplementary material for: Optimizing liver disease screening and surveillance in remote Indigenous Australian communities: the SSOLID study protocol for multi-methodology research
Source: BMJ Open. 2026 Aug 12;16(8):e115620. doi: 10.1136/bmjopen-2025-115620 (PMC13475076; doi:10.1136/bmjopen-2025-115620)
Supplement: Supplementary file 1 [file bmjopen-2025-115620.reviewer_comments.pdf]

PEER REVIEW HISTORY

BMJ Open publishes all reviews undertaken for accepted manuscripts. Reviewers are asked to complete a checklist review form and are provided with free text boxes to elaborate on their assessment. These free text comments are reproduced below.

ARTICLE DETAILS

Title (Provisional)

Optimizing liver disease screening and surveillance in remote Indigenous Australian communities: the SSOLID study protocol for multi-methodology research

Authors

Mackean, Tamara; D'angelo, Shane; Larkin, Michael; Brown, Cath; Pratt, Greg; Valery, Patricia C; Woodman, Richard; Karnon, Jonathan; Medlin, Linda; Riessen, Joshua; Carroll, Melissa; Howell, Jessica; Jeffrey, Gary; Nugent, Michael; Ivanhoe, Terrie; Menzies, Rochelle; Adams, Leon; Bird, Greg; Campbell, Kirsty; Durden, Melanie; Owen, Kelli; Huang, Rae-Lin; Everrett, Iain; Sheppard, Cara; Riessen, Damian; Narayana, Sumudu K; wigg, Alan

---

VERSION 1 - REVIEW

**Reviewer**	**1**
**Name**	**WU, ZHENGYU**
**Affiliation**	**Xiamen University, School of Public Affairs**
**Date**	**18-Mar-2026**
**COI**	**None**

It is a great honor to review this manuscript. The proposed study is highly ambitious, aiming to address the low adherence to liver disease surveillance in remote Indigenous communities in Australia through a stepped-wedge randomized controlled trial combined with Indigenous research methodologies. This approach holds substantial academic value and practical significance in the field of public health, particularly for research involving minority or vulnerable populations.

However, I believe several issues remain to be addressed:

Q1: In extremely remote areas, logistics and staffing pose significant challenges. Although the protocol acknowledges potential risks, the description of contingency plans appears insufficient.

Q2: While the study includes a sample size calculation, participant attrition in remote settings is often higher than anticipated over a multi-year follow-up period, which may ultimately compromise statistical power.

Q3: Given the high likelihood of loss to follow-up in remote areas, it is recommended that the statistical analysis section explicitly specify the sensitivity analyses that will be used to address missing data.

Q4: The diagnostic accuracy of mobile ultrasound devices may vary in uncontrolled environments. Could this affect the fairness of comparisons between the intervention and control groups? It is recommended to provide additional details regarding calibration frequency and quality control procedures for mobile ultrasound equipment under extreme climatic conditions, to ensure the comparability of collected clinical data.

Q5: It is recommended that the ethics section include a discussion of Indigenous data sovereignty, clarifying how data will be stored, who will retain ownership, and how communities will be involved in the interpretation and dissemination of findings.

Q6: In the "on-site monitoring" model, if local expertise is limited, it would be helpful to discuss how digital technologies or telemedicine could be utilized for secondary diagnostic confirmation.

Q7: As a study protocol, it is recommended to include a brief discussion on how the "on-site monitoring" model could be integrated into existing primary healthcare systems after the conclusion of project funding, in order to avoid a situation where services cease once the study ends.

Q8: Please ensure that the terminology used for different types of liver disease throughout the manuscript is consistent with the latest international nomenclature standards.

**Reviewer**	**2**
**Name**	**McMullan, Christel**
**Affiliation**	**University of Birmingham, Institute of Applied Health Research**
**Date**	**02-Apr-2026**
**COI**	**None**

This is a high-quality, ambitious, and ethically strong protocol addressing a major and well-documented health inequity: chronic liver disease and hepatocellular carcinoma (HCC) outcomes for Indigenous Australians living in very remote communities. Here are my comments:

1. Do you have any plans to include Patient and Public Involvement and Engagement throughout the study? If so, could you describe your plans?

2. Yarning circles - you do not mention what type of analysis will be used? Can you add details?

VERSION 1 - AUTHOR RESPONSE

Reviewer: 1
Dr. ZHENGYU WU, Xiamen University
Comments to the Author:
It is a great honor to review this manuscript. The proposed study is highly ambitious, aiming to address the low adherence to liver disease surveillance in remote Indigenous communities in Australia through a stepped-wedge randomized controlled trial combined with Indigenous research methodologies. This approach holds substantial academic value and practical significance in the field of public health, particularly for research involving minority or vulnerable populations.
However, I believe several issues remain to be addressed:

Q1: In extremely remote areas, logistics and staffing pose significant challenges. Although the protocol acknowledges potential risks, the description of contingency plans appears insufficient.
RESPONSE: The Reviewer raised an important point. CIs within the SSOLID team have significant experience with remote area research and have developed the study protocol based on these experiences. We have mitigated some of the risks outlined in the study design as listed below:

- Potential low recruitment is mitigated by providing participant incentive payments at the Liver Check Phase and at each yarning session (Pg 14 and 17).
- Inability to find patients for FibroScan is mitigated by including a Hepascore cut-off of ≥0.8 for entry into the Liver Monitoring Phase (Table 2 on Pg 27).
- Continuous relationship building with the local site staff to ensure they are supported in study conduct, including formal fortnightly meetings with the key study group (Pg 13). These meetings involve discussing recruitment and troubleshooting any particular issues or concerns.
- Lack of buy in from staff mitigated by reasonable payments to each service to cover costs and further payments as part of the Australian Teletrial Program (funding provided per participant recruited). We have now added some information about the Australian Teletrial Program on Pg 13.
- Close liaison with local site staff to ensure study activities and visits occur during optimal times when participants are in community, and factor in cultural events and protocols.
- Support recruitment by creating multi-modal resources (video, visual and printed), translation of resources into local language, study artwork and T-shirts to create a visual representation of the study (Pg 13 and 16).

We have now amended the manuscript to include more information. Furthermore, recruitment for this study has commenced since submission of this manuscript, and we can report that the above strategies have been working well without any significant issues, and the study is on track to meet all recruitment targets at all sites.

Q2: While the study includes a sample size calculation, participant attrition in remote settings is often higher than anticipated over a multi-year follow-up period, which may ultimately compromise statistical power.
RESPONSE: The Reviewer raises an important point regarding participant attrition in remote settings. To address this, we have embedded both operational and design-based mitigation strategies to ensure statistical power is maintained throughout the study:
Proactive Retention through Governance: We have established a robust governance structure, including a First Nations Investigator Group and a Community Leadership Group at each site. These groups, alongside locally recruited Indigenous Project Officers, ensure that

local community needs and cultural practices guide the research conduct. By fostering these strong relationships and maintaining direct communication with participants, we aim to minimize attrition.

Design-Based Engagement: The use of a stepped-wedge RCT design is optimal in this context because it ensures equitable access to care — no participant is excluded from the intervention — which supports sustained participant engagement and reduces the "all-or-nothing" attrition risk associated with traditional parallel cluster trials. The power analysis for this project utilised realistic assumptions, with a 34% adherence rate in the On-Country monitoring group and a 10% drop out rate. As such, the minimum recruitment is 10 participants per site.

Contingency Planning: Furthermore, we are closely monitoring recruitment metrics. Should recruitment fall below the anticipated rate at any point, we have established a contingency plan to extend the recruitment period as necessary to ensure that our original recruitment targets are achieved. Recruitment for this study has commenced since submission of this manuscript and we can confirm that recruitment strategies has been working well and recruitment has exceeded expectations, and as such we are not concerned with impact on our statistical power. This has not been included within the manuscript as it is out of scope.

Q3: Given the high likelihood of loss to follow-up in remote areas, it is recommended that the statistical analysis section explicitly specify the sensitivity analyses that will be used to address missing data.

RESPONSE: We appreciate the reviewer's emphasis on rigorously handling missing data. As our primary analysis is conducted on an Intention-to-Treat (ITT) basis, our statistical plan is specifically designed to address loss-to-follow-up:

Sensitivity Analysis: Missing outcome data will be systematically investigated for patterns, and if assumed to be Missing at Random (MAR), we will conduct a sensitivity analysis using a Generalized Linear Mixed-Effects Model (GLMM) with multiply imputed datasets to ensure our findings remain robust (Pg 18).

As noted in our response to Q2, this statistical approach is complemented by our study design, which utilizes a stepped-wedge RCT that encourages sustained participation by ensuring no community is denied access to care. By combining this design with our active, community-led retention strategies, we are well-positioned to maintain the integrity of our longitudinal data.

Q4: The diagnostic accuracy of mobile ultrasound devices may vary in uncontrolled environments. Could this affect the fairness of comparisons between the intervention and control groups? It is recommended to provide additional details regarding calibration frequency and quality control procedures for mobile ultrasound equipment under extreme climatic conditions, to ensure the comparability of collected clinical data.

RESPONSE: This is a valid point and was considered during the development of this protocol. The study will be using a high-quality mobile ultrasound (Sonosite PX Ultrasound System) which will be calibrated and maintained regularly as per the manufacturer's guidelines. The device is designed to be unaffected by extreme conditions (as per the manufacturer's guide), nor will any sonography visits be conducted in extreme weather conditions. The device will be operated by experienced sonographers in each instance and will be able to conduct on-site troubleshooting and quality control if a need arises. Furthermore, while diagnostic accuracy is important, the primary outcome of this study is on adherence to the On-Country screening rather than diagnostic accuracy.

Q5: It is recommended that the ethics section include a discussion of Indigenous data sovereignty, clarifying how data will be stored, who will retain ownership, and how communities will be involved in the interpretation and dissemination of findings.

RESPONSE: We have amended the manuscript to include a First Nations Data Sovereignty section within the Ethics and Dissemination section. The updated manuscript has been attached to the submission.

Q6: In the "on-site monitoring" model, if local expertise is limited, it would be helpful to discuss how digital technologies or telemedicine could be utilized for secondary diagnostic confirmation.
RESPONSE: The On-Country HCC model is designed to not require local expertise, with a specialist ultrasonographer travelling to each site to conduct the scans. This is to ensure diagnostic accuracy, with each scan being reviewed and reported by a radiologist. All usual care HCC monitoring scans will be done at the closest regional imaging service with a qualified sonographer. The difference between usual care and the On-Country models is regarding the distance participants have to travel to get a scan/access radiology, rather than the quality of the scan. We have now amended the manuscript (Pg 15) to include more information.

Q7: As a study protocol, it is recommended to include a brief discussion on how the "on-site monitoring" model could be integrated into existing primary healthcare systems after the conclusion of project funding, in order to avoid a situation where services cease once the study ends.
RESPONSE: We acknowledge that this is a concern and have a translation and policy working group to focus on translation strategies beyond the scope of this project. Australia has specific funding and infrastructure for Indigenous healthcare and we will connect with these stakeholders to translate study outcomes and sustainably integrate On-Country HCC monitoring into standard practice. We have now amended the manuscript (Pg 20) to include more information.

Q8: Please ensure that the terminology used for different types of liver disease throughout the manuscript is consistent with the latest international nomenclature standards.
RESPONSE: We have reviewed and updated the terminology used within the manuscript to ensure it meets international nomenclature standards. The updated manuscript has been attached to the submission.

Reviewer: 2
Dr. Christel McMullan, University of Birmingham
Comments to the Author:
This is a high-quality, ambitious, and ethically strong protocol addressing a major and well-documented health inequity: chronic liver disease and hepatocellular carcinoma (HCC) outcomes for Indigenous Australians living in very remote communities. Here are my comments:
1. Do you have any plans to include Patient and Public Involvement and Engagement throughout the study? If so, could you describe your plans?
RESPONSE: This has been addressed in the responses to the Editor earlier in this response letter. We have amended the manuscript to include a Patient and Public Involvement Statement on Pg 10.

2. Yarning circles - you do not mention what type of analysis will be used? Can you add details?
RESPONSE: The analysis for the yarning circles involves Indigenous data analysis (yarning analysis as described on Pg 18) rather than qualitative analysis. The data component from yarning is story and our unit of analysis will accordingly be storylines, rather than 'themes'. This type of analysis has been described by Kennedy *et al* (2022) and Siversten *et al* (2025), and supports a decolonising interpretation of research data. We have now amended the manuscript (Pg 18) to include more information.

VERSION 2 - REVIEW

**Reviewer**	**1**
**Name**	**WU, ZHENGYU**
**Affiliation**	**Xiamen University, School of Public Affairs**
**Date**	**13-May-2026**
**COI**	

I think the authors have done a very good job with the revisions and have addressed most of my questions.
