# Supplementary material for: Optimizing liver disease screening and surveillance in remote Indigenous Australian communities: the SSOLID study protocol for multi-methodology research
Source: BMJ Open. 2026 Aug 12;16(8):e115620. doi: 10.1136/bmjopen-2025-115620 (PMC13475076; doi:10.1136/bmjopen-2025-115620)
Supplement: Supplementary file 2 [file bmjopen-16-8-s001.docx]

**PARTICIPANT CONSENT FORM – Liver Monitoring Phase**

| **Project Title** | Optimizing screening and surveillance models of care for liver disease in remote Indigenous Australian communities | |
| --- | --- | --- |
| **Short Title** | SSOLID |  |
| **Chief Investigators** | Professor Alan Wigg and Assoc Professor Tamara Mackean |  |
| **Site Investigators** | Insert details |  |

If you have read the accompanying information sheet and wish to proceed, please sign this consent form.

If you have any further questions, please ask any of the investigators. Please also take the opportunity to discuss this study with your partner / parents and any of the other clinic doctors.

There are 2 parts to this consent form.

**PART 1: Liver Monitoring Study.** By signing below, I am indicating that:

| 1. I understand what this project is about | Yes ☐ | No ☐ |
| --- | --- | --- |
| 2. I voluntarily agree to my participation in this study | Yes ☐ | No ☐ |
| 3. I understand that I can withdraw from the project at any time | Yes ☐ | No ☐ |
| 4. I understand what will happen to me during the research project as explained to me | Yes ☐ | No ☐ |
| 5. I understand that I will be paid for my participation as explained to me | Yes ☐ | No ☐ |
| 6. I understand the potential risks and possible benefits of participating in this research as explained to me | Yes ☐ | No ☐ |
| 7. I understand that the results of this research may be published in a public or other forum | Yes ☐ | No ☐ |
| 8. I understand that all information gathered in this research that is confidential will be kept secure for 15 years | Yes ☐ | No ☐ |
| 9. I want the researcher(s) to give me a copy of the project report that is produced as a result of this research. | Yes ☐ | No ☐ |
| 10. I understand that I will retain any Intellectual Property from my personal interview recordings. | Yes ☐ | No ☐ |
| 11. If testing reveals a condition that requires follow-up medical care, I consent to information being disclosed to [name of Clinic] for that purpose | Yes ☐ | No ☐ |

**Signatures**

Participant to complete:

- I am 18 years of age or older.
- I have read the Participant Information Sheet and Informed Consent Form (or someone has read it to me in language I understand) and I agree with it.

**Name:** __________________________________________

**Signature:** _______________________________________

**Date: /	/**

**Email (to send a copy of this form):** _____________________________

Researcher to complete:

- I have described the nature of the research to the Participant and I believe that he/she understood and agreed to it.

**Name:** __________________________________________

**Signature:** _______________________________________

**Date: /	/**

**PART 2: Liver Monitoring Yarning Circle.** By signing below, I am indicating that:

| 1. I understand what this project is about | Yes ☐ | No ☐ |
| --- | --- | --- |
| 2. I voluntarily agree to my participation in this study | Yes ☐ | No ☐ |
| 3. I understand that I can withdraw from the project at any time | Yes ☐ | No ☐ |
| 4. I understand what will happen to me during the research project as explained to me | Yes ☐ | No ☐ |
| 5. I agree that the researcher(s) can interview me for the research including in a group of people (yarning circle) | Yes ☐ | No ☐ |
| 6. I consent to this interview/focus group/workshop being audio taped on the day | Yes ☐ | No ☐ |
| 7. I understand that I will be paid for my participation as explained to me | Yes ☐ | No ☐ |
| 8. I understand the potential risks and possible benefits of participating in this research as explained to me | Yes ☐ | No ☐ |
| 9. I understand that the results of this research may be published in a public or other forum | Yes ☐ | No ☐ |
| 10. I understand that all information gathered in this research that is confidential will be kept secure for 5 years. | Yes ☐ | No ☐ |
| 11. I want the researcher(s) to give me a copy of the project report that is produced as a result of this research. | Yes ☐ | No ☐ |
| 12. I understand that I will retain any Intellectual Property from my personal interview recordings. | Yes ☐ | No ☐ |

**Signatures**

Participant to complete:

- I am 18 years of age or older.
- I have read the Participant Information Sheet and Informed Consent Form (or someone has read it to me in language I understand) and I agree with it.

**Name:** __________________________________________

**Signature:** _______________________________________

**Date: /	/**

**Email (to send a copy of this form):** _____________________________

Researcher to complete:

- I have described the nature of the research to the Participant and I believe that he/she understood and agreed to it.

**Name:** __________________________________________

**Signature:** _______________________________________

**Date: /	/**

**Form for Withdrawal of Participation**

*Adult providing own consent*

| **Project Title** | Optimizing screening and surveillance models of care for liver disease in remote Indigenous Australian communities | |
| --- | --- | --- |
| **Short Title** | SSOLID |  |
| **Chief Investigators** | Professor Alan Wigg and Assoc Professor Tamara Mackean |  |
| **Site Investigators** | Insert details |  |

**Declaration by Participant**

I wish to withdraw from participation in the above research project and understand that such withdrawal will not affect my routine treatment, my relationship with those treating me or my relationship with Insert site name.

**Signed:**

**Participant’s Name** (printed): **…………………………………………… Date:…………………..**

*In the event that the participant’s decision to withdraw is communicated verbally, the Study Doctor/Senior Researcher will need to provide a description of the circumstances below.*

**Investigator’s Declaration and Signature:**

I have given a verbal explanation of the implications of withdrawal from the research project and I believe that the participant has understood that explanation.

**Name of Investigator** (printed): **………………………………………… Date:…………………..**

Note: All parties signing the consent section must date their own signature.
