# Supplementary material for: Optimizing liver disease screening and surveillance in remote Indigenous Australian communities: the SSOLID study protocol for multi-methodology research
Source: BMJ Open. 2026 Aug 12;16(8):e115620. doi: 10.1136/bmjopen-2025-115620 (PMC13475076; doi:10.1136/bmjopen-2025-115620)
Supplement: Supplementary file 3 [file bmjopen-16-8-s002.docx]

**Participant Information Sheet – Liver Monitoring Phase**

**Interventional Study** - *Adult providing own consent*

| **Project Title** | Optimizing screening and surveillance models of care for liver disease in remote Indigenous Australian communities | |
| --- | --- | --- |
| **Short Title** | SSOLID |  |
| **Chief Investigators** | Professor Alan Wigg and Assoc Professor Tamara Mackean |  |
| **First Nations Investigators** | Assoc Professor Tamara Mackean; Mr Shane D’Angelo; Mr Gregory Pratt; Ms Cath Brown; Mr Michael Larkin; Mrs Linda Medlin; Mr Joshua Riessen; Dr Melissa Carroll; Ms Kelli Owen; Mr Greg Bird |  |
| **Other Investigators** | Prof Patricia Valery; Prof Gary Jeffrey; Dr Sumudu Narayana; Assoc Prof Jessica Howell; Dr Michael Nugent; Ms Terrie Ivanhoe; Assoc Prof Leon Adams; Prof Richard Woodman; Prof Jonathan Karnon; Dr Rae-Lin Huang; Ms Melanie Durden |  |
| **Project Funding** | 2022 MRFF Indigenous Health Research Fund |  |
| **Project Sponsor** | Flinders University |  |

**Introduction:**

We invite you to participate in a study to help us better understand the difficulties patients with liver disease face in remote communities; in order to improve awareness, access to treatment, and care of patients in the future. This information sheet explains why we are performing the study and what is involved should you wish to participate.

This Participant Information Sheet is 5 pages long. Please make sure you have them all. Please read this information carefully and ask questions about anything you don’t understand or want to know more about.

Participating in this research is voluntary. If you don’t wish to take part, you don’t have to. You will receive the best possible care whether or not you take part.

If you decide you want to take part in the research project, you will be asked to sign the consent section. By signing it you are telling us that you:

- Understand what you have read
- Agree to take part in the research project
- Agree to the use of your information as described.

You will be given a copy of this Participant Information Sheet to keep.

**Why are we doing this research?**

The liver is a vital organ that fulfils many functions essential for health such as cleaning toxins from your blood. If the liver becomes hurt, it can lead to inflammation and over time liver scarring (called ‘fibrosis’). Once the liver gets very scarred there are risks of the liver failing and getting a liver cancer.

It is important to check how your liver is doing by getting a liver health check, but we have noticed that getting access to liver care and scans is difficult in remote communities. Mobile technology for liver scans (ultrasound and FibroScan) has changed the way liver care can be delivered to people living in remote areas, especially by removing the need to travel long distances for care.

We are looking to see if providing liver health checks and liver scans using the mobile technology On-Country can improve patients’ health and wellbeing. For example, we are offering liver checks to all participants. We are doing this research trial to make sure that using this mobile technology On-Country is acceptable to you and your community. We are also consulting with your local medical services to find ways we can work better together to improve liver health care.

**Who is organising the research?**

This research project involves a network of liver specialists (hepatologists) and First Nations researchers based in South Australia and Western Australia.

**What does participation involve?**

You will be invited to take part in this research trial if you are found to have an increased risk for liver cancer in the ‘liver check’ study. The Liver Monitoring study continues on from the Liver Check study. As part of this study, each health service taking part in the trial gets randomised. In this study, we are doing a type of randomisation called stepped-wedge, which means that that everyone who is eligible goes into the study and everyone gets both treatments.

If you agree to participate, you will be asked to:

- Undertake a blood test every 6 months (over 2 years) to monitor your liver health. Your local health care worker will collect a blood sample from your arm, and this sample will be used to look for liver disease. This appointment will take approximately 10-15mins.
- Undertake a liver ultrasound every 6 months to monitor your liver health. This will involve the use of new technology that allows ultrasound to be performed On-Country. This scan will take approximately 20mins.

You will have to go to your normal radiology centre some of the time, and the rest of the time you will be able to access the new technology at your local clinic.

- Take part in a yarning circle about your experience with the new technology to manage your liver health. Yarning circles can be one-on-one or be in a small group. The yarn is anticipated to take 1hr and will be held at a place convenient to all participants.
- You will be asked to sign a separate consent form authorising the study to access your Services Australia information, see the separate Services Australia Participant Information Document and Participant Consent Form. Medicare collects information on your medical visits and procedures, and the associated costs, while the PBS collects information on the prescription medications you have filled at pharmacies. The consent form is sent securely to the Department of Human Services who holds this information confidentially. This data will be used to calculate how much the On-Country care model costs compared to the usual care.

Services Australia is not involved in this research other than to provide the information that you have consented to the release of, should you decide to participate in this study. Services Australia has confirmed that this research and any associated documents have received approval from a Human Research Ethics Committee (HREC) that is registered with and operates within guidelines set out by the National Health and Medical Research Council (NHMRC).

You will receive $40 gift certificate if you take part in the yarning circle. Transport to and from the venue can be arranged if required.

**Do I have to take part in this research project?**

Participation in any research project is **voluntary**. If you do not wish to take part, you do not have to. If you decide to take part but later change your mind, you are free to withdraw from the project at any time.

If you do decide to take part, you will be given this Patient Information and Consent Form to sign and you will be given a copy to keep.

Your decision whether or not to take part, or to take part and then withdraw, will not affect your medical treatment or care, your relationship with those treating you.

You may withdraw from the study at any time by telephoning, emailing or writing to us, and filling in a withdrawal of participation form. If you do withdraw your consent during the research project, the research team will not collect additional personal information from you, although personal information already collected will be retained to ensure that the results of the research project can be measured properly and to comply with law. You should be aware that data collected by the research team up to the time you withdraw will form part of the research project results. If you do not want them to do this, you must tell them before you join the research project.

**What are the possible benefits of taking part?**

Participating in this research will have direct benefit to you through access to liver health care On-Country. It is good to get a regular liver health check to look after your liver, especially as it allows for early detection of any liver disease. Information gained from this study is expected to improve the treatment of liver disease in First Nations peoples in the future, especially those living in remote communities.

**What are the possible risks and disadvantages of taking part?**

The information we collect from you will be de-identified so there is no risk to your personal privacy.

Whilst confidentiality will be maintained by the research team and discussed with participants prior to the commencement of the yarning circle, due to the group nature of yarning circles we cannot 100% guarantee your responses will not be disclosed. Discussing liver health, your interactions with health services and broader health experiences may cause some emotional or social distress. If you do experience distress, you can stop participating in the yarning circle at any point. A research team member will provide initial support and offer to assist you to access more specific support with trained staff if you require. You can find the contact details for the team members on Page 4.

If you agree to take part in the study, you will need to provide a blood sample to the researcher(s). This is a routine clinical procedure. Having a blood sample taken may cause some discomfort, bruising, fainting, minor infection or bleeding. If this happens, it can be easily treated. Please let your local medical team know if you experience any issues.

A very rare risk is a false positive from the liver monitoring tests. If a false positive result is identified, you will be referred immediately for scan at your closest radiological centre for further testing. You will also be able to discuss any results with your local doctor in more detail.

**What will happen to information about me?**

Data collected from your medical records will be de-identified and entered into a database under a unique code. This means that researchers using the database on a day-to-day basis will not be able to identify you amongst the group of study participants. The list of unique patient codes will be stored in a separate, secure location by your local health care team, ensuring that your privacy will be protected at all times. By participating in this project, you give us permission to retain and use your clinical data for 15 years. After that information will be deleted from computer files and all paperwork will be shredded.

Your bloods samples collected as part of this study will only be used for standard diagnostic tests to determine if you have liver disease. The samples will be sent to the local pathology centre and only be used for liver disease testing. One sample will be sent to Western Australia to do a specific blood test to see if you have liver scarring, and this test can only be done at a pathology centre in Western Australia. No genetic tests will be done. All samples will be destroyed after 7 days at the pathology centre(s) as per standard requirements.

Yarning circles will be audio recorded and stored securely for five (5) years in a safe, password-protected storage on Flinders University computers. After which they will be electronically destroyed. Only the investigators listed on this information sheet will have access to recordings and written information.

Medical health data collected as part of this study will be shared with your local medical health service for clinic record keeping, and for follow-up purposes.

**What happens when the research project ends?**

When the project ends, the On-Country liver monitoring will also stop. You liver health care will continue with your local health service and any liver monitoring will be go back to the usual processes which may involve travel to the closest radiology centre.

**What will happen to the results of the study?**

During the consent process, you will be given the opportunity to provide your contact details if you wish to receive a copy of the results. If you wish, a one-page summary of the study results and outcomes will be provided to you. Results of the study may also be published in a peer-reviewed journal, presented at conferences or other relevant forums. You can contact the study coordinator Dr Sumudu Narayana on [Sumudu.narayana@sa.gov.au](mailto:Sumudu.narayana@sa.gov.au) or (08) 8204 6421 to receive the results of the study.

**Who is funding the research?**

The study received funding to the value of $2,960,917.80 from the 2022 MRFF Indigenous Health Research Fund grant opportunity. No member of the research team will receive a personal financial benefit from your involvement in this research project (other than their ordinary wages).

**Who has reviewed the research project?**

All research in Australia involving humans is reviewed by an independent group of people called a Human Research Ethics Committee (HREC). The ethical aspects of the project have been approved by the following HRECs:

- Australian Institute of Aboriginal and Torres Strait Islander Studies (AIATSIS) Research Ethics Committee
- Aboriginal Health Research Ethics Committee (AHREC)
- Western Australian Aboriginal Health Ethics Committee (WAAHEC)
- Department of Health WA Human Research Ethics Committee (DOH HREC)
- Southern Adelaide Clinical Human Research Ethics Committee (SAC HREC)

Administrative ethical approval from QIMRB HREC, Central Queensland University HREC, Royal Flying Doctor Service HREC, and the University of Adelaide will also be obtained.

**Further information and who to contact**

The person you may need to contact will depend on the nature of your query.

**Principal Investigator**: [insert site investigator]

| Name |  |
| --- | --- |
| Position |  |
| Telephone |  |
| Email |  |

Other:

| Name | Dr Tamara Mackean |
| --- | --- |
| Position | Associate Professor Aboriginal and Torres Strait Islander Health |
| Telephone | (08) 7221 8467 |
| Email | tamara.mackean@flinders.edu.au |

| Name | Dr Alan Wigg |
| --- | --- |
| Position | Professor, Senior Hepatologist |
| Telephone | (08) 8204 3941 |
| Email | alan.wigg@sa.gov.au |

| Name | Dr Sumudu Narayana |
| --- | --- |
| Position | Trial Coordinator |
| Telephone | (08) 8204 6421 |
| Email | sumudu.narayana@sa.gov.au |

For matters relating to research at the site at which you are participating, the details of the local site complaints person are:

**Local Complaints contact person**

| Name |  |
| --- | --- |
| Position |  |
| Telephone |  |
| Email |  |

If you have any questions or complaints about any aspect of the project, the way it is being conducted or any questions about being a research participant in general, then you may contact:

| Reviewing HREC name | AIATSIS Research Ethics Committee |
| --- | --- |
| HREC Executive Officer | Executive Director of Research, AIATSIS |
| Email | ethics@aiatsis.gov.au |

**Reviewing HREC approving this research** **and HREC Executive Officer details**

- The Chair of the AIATSIS Research Ethics Committee, AIATSIS, 51 Lawson Crescent, Acton ACT 2601, (e) [ethics@aiatsis.gov.au](mailto:ethics@aiatsis.gov.au)
- If I think there has been a breach of my privacy I can write to the Office of the Australian Information Commissioner, GPO Box 5218 Sydney NSW 2001 or call 1300 363 992.

**Local HREC approving this research** **and HREC Executive Officer details**

| Name |  |
| --- | --- |
| Position |  |
| Telephone |  |
| Email |  |
